# The “Pacman Flap with Tongue” for Secondary Orbital Reconstruction in Osteoradionecrosis: A Case Series

**DOI:** 10.3390/medicina62030607

**Published:** 2026-03-23

**Authors:** Michał Gontarz, Piotr Idzi, Katarzyna Egelhoff, Jakub Bargiel, Krzysztof Gąsiorowski, Kamil Nelke, Grażyna Wyszyńska-Pawelec

**Affiliations:** 1 Department of Cranio-Maxillofacial Surgery, University Hospital, 30-688 Cracow, Poland; kegelhoff@su.krakow.pl (K.E.); jakub.bargiel@uj.edu.pl (J.B.); krzysztof.gasiorowski@uj.edu.pl (K.G.); grazyna.wyszynska-pawelec@uj.edu.pl (G.W.-P.); 2 Department of Cranio-Maxillofacial Surgery, Jagiellonian University Medical College, 31-008 Cracow, Poland; 3 Department of Digital Presentation and Creation Techniques, Faculty of Sculpture, Jan Matejko Academy of Fine Arts in Krakow, 31-157 Cracow, Poland; pidzi@asp.krakow.pl; 4 Maxillo-Facial Surgery Ward, EMC Hospital, Pilczycka 144, 54-144 Wrocław, Poland

**Keywords:** orbital exenteration, orbital reconstruction, osteoradionecrosis, temporalis muscle flap, scalp flap, facial epithesis, maxillofacial prosthetics, CBCT, three-dimensional printing

## Abstract

*Background and Objectives*: Orbital exenteration performed for advanced malignancies often results in complex defects that are difficult to reconstruct, particularly in patients treated with adjuvant radiotherapy who subsequently develop osteoradionecrosis. This study describes the preliminary results of a surgical technique for secondary orbital reconstruction using a combined scalp flap and temporalis muscle flap (TMF), referred to as the “Pacman flap with tongue,” performed prior to prosthetic rehabilitation. *Materials and Methods*: Five elderly patients with multiple comorbidities and osteoradionecrosis following orbital exenteration and radiotherapy underwent secondary orbital reconstruction using the “Pacman flap with tongue” technique. The clinical outcomes, flap viability, complications, and feasibility of subsequent prosthetic rehabilitation were assessed. After stabilization of healing, digitally planned ocular epitheses were fabricated using cone-beam computed tomography (CBCT), computer-aided design, and three-dimensional printing. *Results*: Healing was uneventful in all patients. No flap necrosis, wound dehiscence, or recurrent bone exposure was observed. The reconstructed orbital sockets provided a stable, well-vascularized prosthetic bed, enabling satisfactory prosthetic rehabilitation. *Conclusions*: The “Pacman flap with tongue” may be considered a feasible option for secondary orbital reconstruction in selected high-risk patients, particularly in the setting of osteoradionecrosis.

## 1. Introduction

Extensive maxillofacial defects have long posed a significant reconstructive challenge due to the involvement of multiple and diverse tissue types, including skin, muscle, bone, cartilage, and mucosa, as well as complex anatomical structures such as the auricle, orbit, nose, and oral cavity. Restoration of lost tissues and organs is of critical functional and esthetic importance, as it significantly improves patients’ self-esteem and overall quality of life. Complex maxillofacial defects may be reconstructed surgically, prosthetically using an epithesis, or through a combined approach [1,2]. Typical facial defect sites managed with epitheses include the orbit, nose, and auricle.

The most common indications for orbital exenteration are skin malignancies, including basal cell carcinoma, squamous cell carcinoma, and malignant melanoma. Moreover, many patients undergoing extensive oncologic resection require adjuvant radiotherapy, which adversely affects tissue healing and may lead to osteoradionecrosis [3,4,5,6]. Surgical reconstruction of extensive maxillofacial defects is often a multistage process and is associated with the risk of morbidity at both donor and recipient sites [5,6,7]. In addition, orbital defects can be reconstructed using regional flaps, such as the temporalis muscle flap (TMF), forehead flap, or galeal flap, or with free flaps, including the anterolateral thigh flap (ALT) and the free radial forearm flap (FRFF) [3,4,5,6,7,8]. However, achieving satisfactory surgical reconstruction of the orbit remains challenging due to the absence of the eyeball and eyelids. Another major limitation of surgical reconstruction in extensive maxillofacial defects is the time required, including prolonged hospitalization and, often, multiple admissions resulting from multistage surgical procedures, which are associated with an increased risk of postoperative complications [6]. Moreover, most oncological patients requiring reconstruction are elderly and present with multiple comorbidities, further increasing the risk of complications related to general anesthesia.

Custom-made facial prosthetics represent an alternative approach to the reconstruction of maxillofacial defects [1]. Prosthetic rehabilitation may be particularly suitable for patients in whom successful surgical reconstruction is not feasible due to factors such as extensive orbital defects, poor prognosis, significant comorbidities, impaired healing related to scarring, or failure of previous reconstructive procedures. Orbital reconstruction with an epithesis is a primary method of rehabilitation for patients following orbital exenteration, most often due to cancer [8,9]. This approach provides an effective means of improving facial esthetics while remaining minimally invasive. The primary objectives of this procedure are threefold: to replace the missing orbital tissues following resection, to restore facial symmetry, and to enhance appearance. These objectives are important for both the patient’s quality of life and their social acceptance. A further benefit of this approach is the straightforward access it affords to the postoperative site, thus facilitating regular oncological surveillance and early detection of potential recurrence, given prosthesis removal is easy to handle [8,9,10].

The most favorable esthetic outcomes following orbital exenteration are achieved through a combination of surgical orbital socket reconstruction and prosthetic rehabilitation using an epithesis [8,9,10]. Various methods for prosthesis retention have been described, ranging from simple to technologically advanced solutions. The most straightforward and widely available method involves the use of adhesive glues, which do not require additional surgical procedures and are cost-effective. However, this approach is associated with limited stability, a risk of skin irritation, and the need for daily application and removal of the prosthesis. Mechanical attachment to eyeglasses is less commonly employed [9,10].

Currently, bone-anchored titanium implants are considered the gold standard for orbital prosthesis stabilization. In this approach, titanium implants are placed in the orbital rim, and the prosthesis is retained using magnetic attachments or clips. This method provides superior stability, improved patient comfort, and predictable esthetic outcomes [10,11]. Nevertheless, it requires additional surgical procedures and carries inherent risks, including peri-implant infection and complications related to osseointegration, particularly in patients who have undergone radiotherapy [12].

Orbital reconstruction poses a particular challenge in patients with osteoradionecrosis, which may result in exposure of the orbital rim bone [13]. In such cases, implant-retained prostheses alone are contraindicated due to the increased risk of complications and progression of osteoradionecrosis. These patients require sequestrectomy followed by reconstruction using well-vascularized regional or free flaps to provide a stable and adequately perfused prosthetic bed for subsequent epithesis placement [14].

The present study describes preliminary results of a surgical technique for secondary orbital reconstruction in patients with osteoradionecrosis, employing a scalp flap combined with a temporalis muscle flap (TMF), referred to as the “Pacman flap with tongue,” performed as a preparatory procedure prior to prosthetic rehabilitation.

## 2. Materials and Methods

Between August 2024 and August 2025, five patients underwent secondary orbital reconstruction using the “Pacman flap with tongue” technique at the Department of Cranio-Maxillofacial Surgery, Jagiellonian University in Kraków. This retrospective case series included all consecutive patients meeting the inclusion criteria during the study period. All patients were elderly and presented with multiple comorbidities as well as osteoradionecrosis. Each patient had previously undergone orbital exenteration followed by spontaneous granulation and postoperative radiotherapy, which subsequently resulted in bone exposure and necessitated secondary reconstruction. The patients’ characteristics are summarized in Table 1.

### 2.1. Surgical Technique

**Figure 1:**
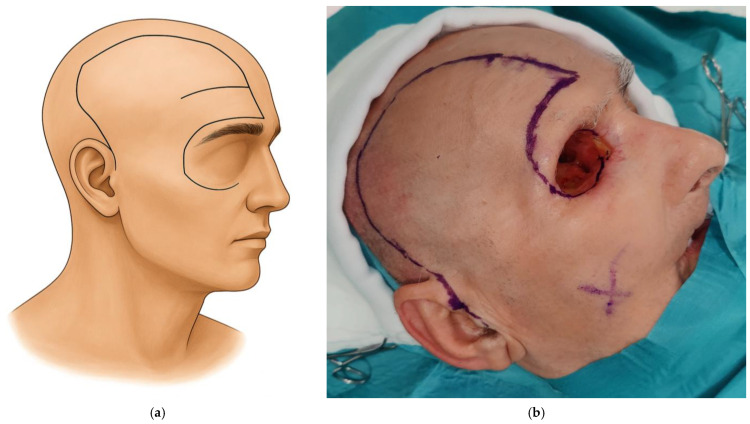
Schematic illustration of the scalp flap marked from the frontal crest above the eyebrow to the midline, following a trajectory similar to the pterional approach and extending in a preauricular direction (**a**). Clinical intraoperative view showing the marked incision lines (**b**).

The scalp flap is designed according to the size of the orbital defect requiring coverage. Care is taken to align the flap design with the position of the eyebrow to achieve a natural final appearance. The flap is marked from the frontal crest above the eyebrow to the midline and follows a trajectory similar to the pterional approach, extending in a preauricular direction (Figure 1). Once the design is completed, an incision is made through the skin, subcutaneous tissue, and galea. The scalp flap is then elevated in the loose areolar plane just above the pericranium, temporal fascia, and temporalis muscle.

**Figure 2:**
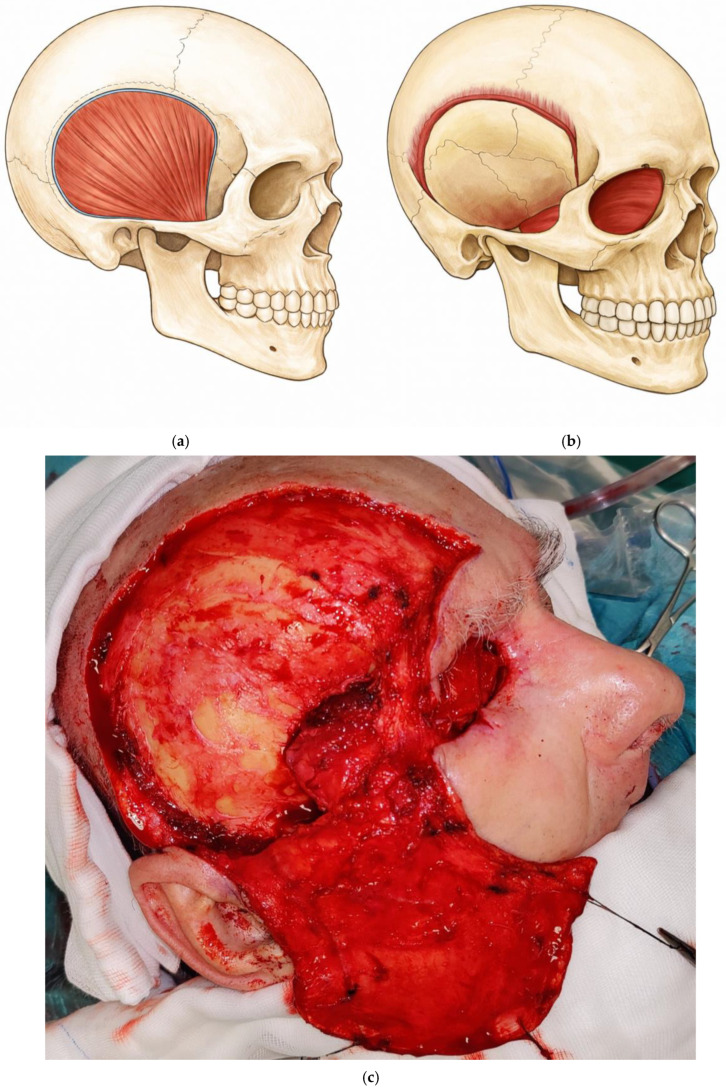
Schematic illustration of the deep operative field above the pericranium, temporal fascia, and temporalis muscle after elevation of the scalp flap (**a**). Schematic illustration of the dissected temporalis muscle, rotated and inset to fill the orbital defect and secured with sutures (**b**). Clinical intraoperative view showing the elevated scalp flap with the dissected temporalis muscle rotated to fill the orbital defect (**c**).

Dissection of the temporalis muscle flap (TMF) is performed using Colorado-tip electrocautery, beginning just posterior to the junction of the zygomatic process of the frontal bone and the frontal process of the zygomatic bone, and proceeding superiorly and posteriorly to elevate the temporalis muscle. After division at its origin, the TMF is raised using a broad periosteal elevator, exposing the underlying cranial bones. Particular care is taken during this step to avoid excessive traction or torsion of the deep temporal vessels, as this could compromise flap perfusion. A burr is then used to open the orbit through the lateral orbital wall, preserving the orbital margin to facilitate tension-free insertion of the TMF into the orbital cavity. The harvested TMF is subsequently rotated, inset to fill the orbital defect, and secured with sutures (Figure 2).

**Figure 3:**
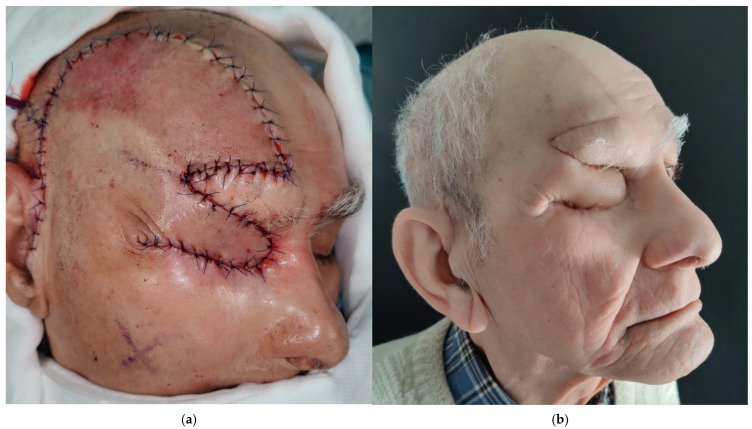
Clinical view showing the final intraoperative result of orbital reconstruction using the “Pacman flap with tongue” (**a**). Clinical result 4 weeks after surgery, showing visible flap bulkiness and a dog ear deformity in the lower aspect of the scar in the zygomatic region, without wound dehiscence or orbital bone exposure (**b**).

Following orbital filling with the TMF, the scalp flap is divided into two components. The inferior portion, approximately 3 cm in width, is rotated and transferred to the orbital defect to cover the TMF (Figure 3). After both flaps are positioned and sutured, an active drain is placed beneath the scalp flap, and the temporal region is dressed with a mild compression bandage.

### 2.2. Prosthetic Manufacturing

At least three months after surgical reconstruction, once postoperative edema had resolved and flap shrinkage had stabilized, the prosthetic manufacturing process was initiated. The process began with a prosthetic consultation and a comprehensive clinical evaluation of the post-exenteration orbital region, including assessment of the reconstructive flap used to restore the defect. Cone-beam computed tomography (CBCT; CS 9600 CBCT Scanner, Atlanta, GA, USA) was performed to obtain detailed anatomical data for subsequent digital processing. Based on the CBCT data, a three-dimensional digital model of the patient’s face was created. Using digital sculpting software (3D Slicer, 5.10.0), the ocular epithesis was virtually designed to achieve anatomical accuracy and esthetic harmony with the patient’s facial features.

A resin-based 3D printer (Formlabs Form 3b, Formlabs, Somerville, MA, USA) was then used to fabricate both the facial model and the digitally designed prosthesis. These printed models enabled evaluation of prosthesis dimensions and their relationship to surrounding structures. Due to the rigidity and limited color fidelity of resin materials, the printed prototype served primarily for trial fitting and as a reference for mold fabrication. This step allowed for precise adjustments to ensure optimal adaptation to the patient’s soft tissues.

**Figure 4:**
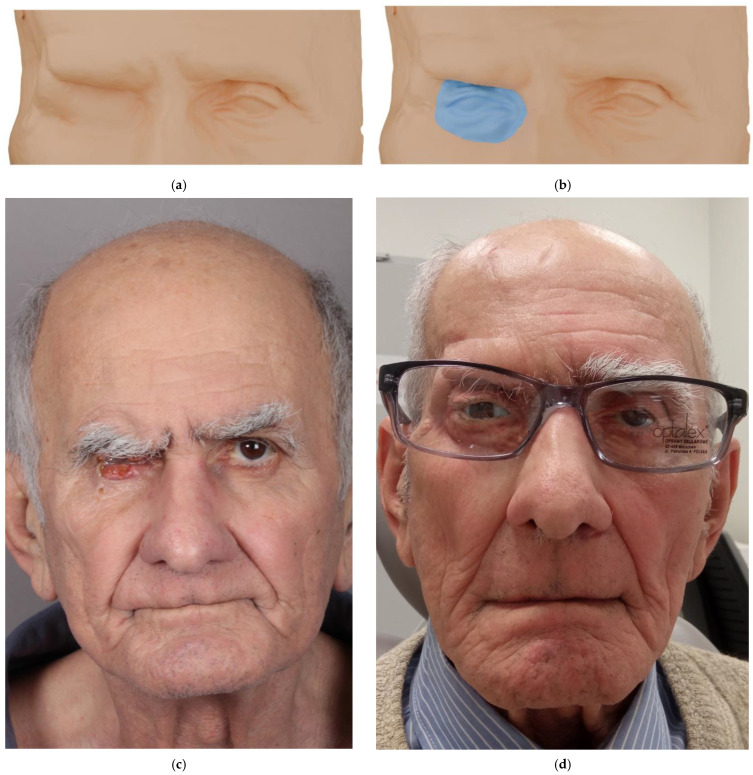
Facial scan based on CBCT showing the reconstructed orbital defect using the “Pacman flap with tongue” four months after surgery (**a**). CBCT-based scan showing a virtually designed ocular epithesis created to achieve anatomical accuracy and esthetic harmony with the patient’s facial features, based on mirror imaging of the healthy contralateral side (**b**). Clinical view of a patient with basal cell carcinoma of the lower eyelid with orbital infiltration before surgical treatment (**c**). Final clinical result in a patient who underwent right orbital exenteration due to basal cell carcinoma, postoperative radiotherapy complicated by osteoradionecrosis, sequestrectomy, and secondary reconstruction using the “Pacman flap with tongue,” with ocular epithesis attached to eyeglasses. A satisfactory esthetic result is visible, with hollowing in the right temporal region (**d**).

The definitive prosthesis was cast in medical-grade silicone (Platsil Gel 10, fast-curing platinum/addition cure) and meticulously pigmented using an airbrush system (Nebula Airbrush make-up set) to replicate the translucency and coloration of the patient’s natural skin. Anatomical details such as vascular patterns, subtle discolorations, and eyelashes were incorporated to enhance realism. The ocular globe was produced on a digitally modeled shell corresponding precisely to the natural ocular curvature. A high-resolution image of the patient’s healthy eye—accurately representing iris color, vascular structures, and other unique features—was digitally applied to the surface. The customized ocular shell was then manufactured using PolyJet printing technology (Stratasys J5 MediJet), finished, and integrated into the silicone prosthesis (Figure 4).

## 3. Results

The postoperative healing course was uneventful in all patients. Sutures were removed 10–14 days after surgery. No cases of flap necrosis, wound dehiscence, or recurrent orbital bone exposure were observed. Transient palsy of the frontal branch of the facial nerve occurred in all patients and resolved spontaneously within a mean period of three months. Minor secondary revision for correction of a dog-ear deformity in the zygomatic region was required in all cases and was performed on an outpatient basis under local anesthesia at least four weeks after the primary reconstruction. During a mean follow-up period of 11.4 months, no recurrence of osteoradionecrosis was observed.

## 4. Discussion

The fabrication of an ocular epithesis aims to achieve both esthetic and functional reconstruction of the orbital defect following surgical exenteration. The prosthesis restores the natural appearance of the periocular region, improves facial symmetry, and significantly enhances the patient’s psychosocial well-being. The procedure integrates diagnostic imaging, digital modeling, and additive manufacturing techniques to ensure high precision and individualization of the final outcome. Preparation of the prosthetic bed prior to rehabilitation is crucial for achieving a satisfactory esthetic result. In patients who have not undergone orbital reconstruction following exenteration and whose healing has occurred through spontaneous granulation after postoperative radiotherapy, complications such as orbital bone exposure are observed more frequently [14,15,16].

Prosthetic rehabilitation in this group of patients is particularly challenging for several reasons. Direct contact between the epithesis and orbital tissues exerts pressure on the underlying substrate, thereby increasing the risk of bone exposure. Achieving stable retention of the prosthesis is also difficult. The use of adhesives may irritate orbital tissues, further predisposing patients to bone exposure. Similarly, the use of osseointegrated implants to improve prosthesis stability carries a risk of inducing osteoradionecrosis, potentially leading to implant loss. Consequently, these patients require prosthetic rehabilitation only after appropriate preparation of the prosthetic bed to minimize the risk of bone exposure associated with osteoradionecrosis. In such cases, secondary reconstruction using well-vascularized flaps provides a reliable solution, promoting proper healing and reducing the likelihood of recurrent bone exposure [5,15,17]. Both free flaps and TMF are commonly employed for this purpose [3,17,18,19].

In most reported cases of orbital reconstruction using TMF, the external surface is covered with a skin graft [3,20,21]. However, the healing process in such cases can be unpredictable, and skin graft necrosis may occur, potentially exacerbating inflammation and increasing the risk of bone exposure [3]. To minimize this risk, the present study describes an original method of orbital reconstruction using two flaps, referred to as the “Pacman flap with tongue”. In this technique, the TMF fills the deep portion of the orbit, while a scalp flap reconstructs the external surface, promoting a more predictable blood supply and a rapid healing process. Furthermore, given the history of prior osteoradionecrosis, the use of implants to improve prosthesis retention was avoided to reduce the risk of bone re-exposure. Because the orbital cavity was filled with two flaps, spontaneous prosthesis retention was not feasible; therefore, the epithesis was stabilized using eyeglasses.

**Figure 5:**
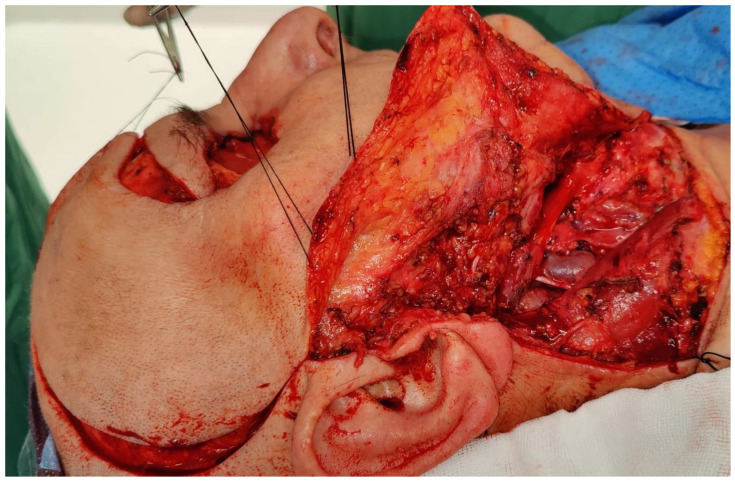
Intraoperative view showing right orbital exenteration with formation of a “Pacman flap with tongue,” combined with therapeutic right parotidectomy and selective neck dissection (levels II and III, with adjunct to the external jugular vein).

This report describes our institutional experience with a combined scalp–temporalis flap configuration applied in patients with osteoradionecrosis prior to prosthetic rehabilitation. The “Pacman flap with tongue” may offer potential benefits in selected cases, particularly in complex cases following tumor resection or in patients with osteoradionecrosis and inflammatory conditions associated with secondary healing by granulation. In our case series, the vascularized nature of the combined flaps was associated with satisfactory flap survival and adequate orbital coverage. This technique enables one-stage reconstruction, which can be combined with therapeutic parotidectomy and neck dissection (Figure 5), and it may also be used after craniectomy to reduce the risk of cerebrospinal fluid leakage [22].

Compared with microsurgical reconstruction, this regional flap approach may reduce operative complexity in selected patients, with many patients discharged within two days postoperatively. However, formal cost and time analyses were not performed in this study. Unlike microsurgical reconstructions, it does not require specialized equipment. In addition, the texture and color of scalp skin closely resemble adjacent tissues, and some patients may be satisfied with this reconstruction without the need for prosthetic rehabilitation. Nevertheless, potential complications must be considered. Temporal hollowing and transient palsy of the temporal branch of the facial nerve have been reported, and some patients may experience mastication difficulties [3,20]. Additionally, secondary correction of a “dog’s ear” deformity may be required, although this can be easily performed under local anesthesia in an outpatient setting. Overall, the “Pacman flap with tongue” may represent a reliable and versatile option for orbital reconstruction, balancing surgical efficiency with predictable functional and esthetic outcomes.

The innovation and adaptation of digital technologies not only accelerate and streamline workflows in prosthodontic and maxillofacial surgery departments but, most importantly, provide tangible benefits for patients. In the present study, the epithesis was designed based on CBCT imaging. CBCT enables rapid acquisition of high-quality digital facial data, eliminating the need for conventional impressions and reducing patient discomfort and stress. Moreover, digital sculpting software for the manipulation of complex three-dimensional datasets offers virtually unlimited possibilities in maxillofacial prosthetic reconstruction. The resulting ocular epithesis demonstrates high anatomical and esthetic fidelity, providing accurate adaptation to facial tissues and a lifelike replication of the natural eye. The integration of digital imaging, computer-aided design, and three-dimensional printing technologies enables a fully individualized and reproducible workflow, contributing to superior esthetic outcomes and improved quality of life following orbital exenteration. Nevertheless, high-resolution 3D printers and resin materials still require further investigation, particularly regarding biocompatible elastic resins that may eventually replace silicone and allow direct 3D printing of restorative prostheses. The continued development of digital technologies may help reduce costs, improve quality and efficiency, and overcome technical barriers that currently limit access to facial prostheses for many patients worldwide.

This study has several limitations. The follow-up period was relatively short, limiting the assessment of long-term outcomes and reconstruction durability. In addition, the retrospective design may introduce inherent biases and limits the ability to establish causal relationships. This retrospective case series does not include standardized functional or esthetic outcome measures, validated patient-reported quality-of-life assessments or objective grading of complications. Furthermore, the study was conducted at a single center and included a small number of patients, which may restrict the generalizability of the findings to broader populations. Future studies involving larger patient cohorts and longer follow-up periods, with comparison of cost-effectiveness against free flap reconstruction, are required to validate the reproducibility, long-term stability, and complication profile of the “Pacman flap with tongue” technique.

## 5. Conclusions

The “Pacman flap with tongue” may represent an alternative method for orbital reconstruction in cases of osteoradionecrosis following radiotherapy and serves as a viable option to free flap reconstruction. This technique represents a potential reconstructive option for secondary orbital reconstruction in patients unsuitable for microsurgical reconstruction. The “Pacman flap with tongue” may offer predictable vascularity, sufficient bulk for complete orbital coverage, and the possibility of one-stage reconstruction, even after tumor resection or in the setting of osteoradionecrosis. Additionally, the technique does not require microsurgical expertise and may be considered in elderly or comorbid patients, although further studies are required to evaluate recovery profiles and cost-effectiveness.

## Figures and Tables

**Table 1:** Demographic and clinical characteristics of the study population.

Case	Age/Sex	Primary Cancer	Orbit Healing	Neck Dissection	Reconstruction After Radiotherapy (Months)	Follow Up (Months)
1	81/M	SCC	Spontaneous granulation	No	21	14/NED
2	77/M	MCC	Spontaneous granulation	TP + SND	10	18/NED
3	76/F	MCC	Spontaneous granulation	TP + SND	9	6/DOD
4	68/M	BCC	Spontaneous granulation	No	7	8/NED
5	74/M	SCC	Spontaneous granulation	No	13	11/NED

M—male, F—female, SCC—squamous cell carcinoma, MCC—Merkel cell carcinoma, BCC—basal cell carcinoma, TP—therapeutic parotidectomy, SND—selective neck dissection, NED—no evidence of disease, DOD—death of disease.

## Data Availability

The data were obtained from patients operated on the Cranio-Maxillo-Facial Surgery Department of Jagiellonian University, Cracow, Poland, and cannot be shared in accordance with the General Data Protection Regulation (EU) 2016/679.

## Abbreviations

The following abbreviations are used in this manuscript:

TMF	Temporalis muscle flap
ALT	Anterolateral thigh flap
FRFF	Free radial forearm flap
CBCT	Cone-beam computed tomography

## References

[1] Egelhoff K., Idzi P., Bargiel J., Wyszyńska-Pawelec G., Zapała J., Gontarz M. (2022). Implementation of cone beam computed tomography, digital sculpting, and three-dimensional printing in facial epithesis—A technical note. Appl. Sci..

[2] Nyberg E.L., Farris A.L., Hung B.P., Dias M., Garcia J.R., Dorafshar A.H., Grayson W.L. (2017). 3D-printing technologies for craniofacial rehabilitation, reconstruction, and regeneration. Ann. Biomed. Eng..

[3] Uyar Y., Kumral T.L., Yıldırım G., Kuzdere M., Arbağ H., Jorayev C., Kılıç M.V., Gümrükçü S.S. (2015). Reconstruction of the orbit with a temporalis muscle flap after orbital exenteration. Clin. Exp. Otorhinolaryngol..

[4] Gąsiorowski K., Gontarz M., Marecik T., Szczurowski P., Bargiel J., Zapała J., Wyszyńska-Pawelec G. (2024). Risk factors for orbital invasion in malignant eyelid tumors: Is orbital exenteration still necessary?. J. Clin. Med..

[5] Philips R., Topf M.C., Graf A., Krein H., Heffelfinger R., Luginbuhl A., Curry J. (2019). Orbital outcomes after orbit-sparing surgery and free flap reconstruction. Oral Oncol..

[6] Gąsiorowski K., Gontarz M., Bargiel J., Marecik T., Szczurowski P., Wyszyńska-Pawelec G. (2024). Reconstructive techniques following malignant eyelid tumour excision—Our experience. J. Clin. Med..

[7] López F., Suárez C., Carnero S., Martín C., Camporro D., Llorente J.L. (2013). Free flaps in orbital exenteration: A safe and effective method for reconstruction. Eur. Arch. Otorhinolaryngol..

[8] Kuehnel S., Grimm A., Bohr C., Hosemann W., Weber R., Ettl T., Kuehnel T. (2023). Reconstruction of the exenterated orbit with an island pericranial flap: A new surgical approach. Plast. Reconstr. Surg. Glob. Open.

[9] Ritschl L.M., Schwarz M., Klinger F., Wolff K.D., Niu M., Weitz J. (2024). Extended orbital exenteration, epithetic restoration, and patient supply: A cross-sectional study of a historic cohort. Head Neck.

[10] Vincent A., Kohlert S., Kadakia S., Sawhney R., Ducic Y. (2019). Prosthetic reconstruction of orbital defects. Semin. Plast. Surg..

[11] Eo M.Y., Cho Y.J., Nguyen T.T.H., Seo M.H., Kim S.M. (2020). Implant-supported orbital prosthesis: A technical innovation of silicone fabrication. Int. J. Implant. Dent..

[12] Granström G., Tjellström A., Brånemark P.I., Fornander J. (1993). Bone-anchored reconstruction of the irradiated head and neck cancer patient. Otolaryngol. Head Neck Surg..

[13] Peter N.M., Laitt R., Leatherbarrow B. (2014). Osteoradionecrosis of the exenterated orbit. Orbit.

[14] Martel A., Baillif S., Nahon-Esteve S., Gastaud L., Bertolotto C., Lassalle S., Lagier J., Hamedani M., Poissonnet G. (2021). Orbital exenteration: An updated review with perspectives. Surv. Ophthalmol..

[15] Jategaonkar A.A., Vernon D., Byrne P.J. (2019). Regional Reconstruction of Orbital Exenteration Defects. Semin. Plast. Surg..

[16] Yesensky J., Lebo N. (2020). Reconstructive options following orbital exenteration. Curr. Opin. Otolaryngol. Head Neck Surg..

[17] Ein L., Daniyan O., Nicolli E. (2019). Temporalis muscle flap. Oper. Tech. Otolaryngol. Head Neck Surg..

[18] Bottini G.B., Joos V., Steiner C., Zeman-Kuhnert K., Gaggl A. (2024). Advances in Microvascular Reconstruction of the Orbit and Beyond: Considerations and a Checklist for Decision-Making. J. Clin. Med..

[19] Kesting M.R., Koerdt S., Rommel N., Mücke T., Wolff K.-D., Nobis C.P., Ringel F., Frohwitter G. (2017). Classification of orbital exenteration and reconstruction. J. Craniomaxillofac. Surg..

[20] Lam D., Carlson E.R. (2014). The temporalis muscle flap and temporoparietal fascial flap. Oral Maxillofac. Surg. Clin. N. Am..

[21] Torroni A., Cervelli D., Gasparini G., Grussu F., Moro A., Marianetti T.M., Foresta E., Azzuni C., Pelo S. (2015). Anterior retrograde approach to the myofascial temporalis muscle for orbital reconstruction: Series of 9 consecutive cases. Ann. Plast. Surg..

[22] Sahni M., Patel P., Lakhera K.K., Singh S., Sharma R. (2023). Use of temporalis muscle and temporoparietal fasciocutaneous flap (TPPF) for orbital exenteration defects: Our experience of 10 cases. Indian J. Otolaryngol. Head Neck Surg..

